# A Reminder From the Devastation Hurricane Maria Left Behind

**DOI:** 10.7759/cureus.2038

**Published:** 2018-01-08

**Authors:** Angel Lopez-Candales, Dagmar F Hernandez-Suarez, Anthony D Osterman-Pla, José G Conde-Santiago

**Affiliations:** 1 Medicine, University of Puerto Rico School of Medicine

**Keywords:** floods, catastrophe, cardiovascular diseases, heath, pollution

## Abstract

On September 20, 2017, Hurricane Maria hit Puerto Rico, causing an unprecedented humanitarian crisis on a level that none of us have experienced before. The following editorial intends to show a physician's perspective of the impact of this storm on healthcare, particularly in triggering cardiovascular events.

## Editorial

Despite great debates about whether climate warming is real, global weather patterns have already changed, and storms are expected to become more violent. Flood hazards are a major threat to many parts of the world and the Caribbean is no exception. In fact, floods are the most frequent natural disasters in this region.

Hurricane Maria, a Category 4 hurricane, made landfall in Puerto Rico on September 20th, 2017. The right elements for a “perfect storm” came about as the island was just recovering from the effects of another storm. Two weeks earlier, Irma, a Category 5 hurricane, had brushed off the northeastern portion of Puerto Rico, causing a partial collapse of the island’s power grid as well as an overall sense of instability. The true apocalyptic scenario shared by some 3.5 million people trapped within the confines of an island (100 miles by 35 miles) came as the population was largely unprepared and still crippled from the previous storm. Unfortunately, the much-feared and now evidently frail infrastructure, as well as the pre-storm handicapped economy from an ongoing financial crisis, left a vibrant island in total chaos. As the 200 mph winds calmed down and floods began to recede, Puerto Ricans were looking at each other in disbelief and were frightened. Most of the population was isolated from each other as a result of collapsed bridges, inoperable roads, and almost total lack of telecommunications. In fact, for days, the faint voice of only one radio station remained; widespread lack of power, limited availability of drinking water, food supplies, medications, and fuel reserves added to an overall sense of desolation.

Though the magnitude of personal property and material loss are difficult to describe unless they are witnessed firsthand, the extent of human tragedy and suffering is simply heartbreaking and rightly classified as a humanitarian crisis. However, as Franklin D. Roosevelt once said during his inaugural address, “the only thing we have to fear is fear itself”. The wisdom behind this expression never felt so real until now, and these are words that truly motivated all left standing. Indeed, we all felt fear as well as other concerns of not hearing from loved ones and so many other uncertainties, leaving no time to go through the usual stages of grief and loss.

Sure, all health care providers will recognize trauma, injuries, and drowning as immediate, direct threats from hurricanes. Following these waves of injuries, different kind of infections take center stage and begin to flourish in the immediate days after such a disaster, particularly due to poor sanitation and other water-borne diseases likely to affect the poor and vulnerable. In addition, infection hazards are further aggravated by ingestion of spoiled food due to flooding or power failure, contaminated drinking water, spikes in mosquito populations, rodents, dead animals, as well as the widespread growth of mold and mildew [1]. Lastly, indirect health hazards also occur during cleanup activities as individuals may encounter fallen power lines, gas leaks, and countless debris from damaged poles, trees, roads, bridges as well as artifacts that remain submerged in water; all of which could be fatal or result in significant injury or open wounds prone to infection.

Additional concerns also center on the potential for these natural disasters to cause severe anxiety and post-event depression and how these can trigger acute mental health issues. Both of these entities interfere with individuals taking scheduled maintenance medications and other therapies, with the potential of destabilizing chronic conditions and creating acute medical emergencies. Additionally, anxiety and panic attacks coupled with unusual physical effort may lead to acute cardiovascular (CV) events.

Though data regarding the magnitude of cardiovascular events as a result of tsunamis is quite convincing [2]; data secondary to flooding is scarce. In this regard, Vanasse and associates published their results on acute CV events after the spring flood of 2011 that occurred on Saint-Jean-sur-Richelieu [3]. Even though 27% more acute CV events were identified during that period when compared to the same period in 2010 and 2012, the association was not statistically significant. Potential limitations were the small number of individuals affected; the relatively minor extent of the flood and that access to health care service was not affected. In contrast, Puerto Rico might offer a unique setting to prove this hypothesis since all these previous limitations will no longer be applicable.

It is well known that a significant, acute stressful situation has the potential of causing derangements of the sympathetic nervous system and hypothalamic-pituitary-adrenal gland axis. Clinically relevant consequences of such events include uncontrolled diabetes, hypertension, destabilization of common respiratory conditions, development of acute anxiety, panic attacks, and delirium [2]. All of these clinical scenarios are by themselves triggers of CV events such as unstable angina, myocardial infarction, Takotsubo cardiomyopathy, ventricular arrhythmias - with or without risk for sudden cardiac death, pulmonary edema, decompensated heart failure, and cerebrovascular accidents.

As we continue to struggle and improvise on ways to regain some normalcy, let’s not lose sight of the bigger picture. The road to recovery will not be easy and many hardships are expected in the coming days, weeks, and months. Once this mayhem is over and all the help is gone, we might be more vulnerable than we are today. Our already frail health care system might not be able to withstand the potential onslaught of patients with chronic conditions that may destabilize as well as those that could present with acute CV events. Even though the great catastrophe will be long gone, the scars left behind will take a long time to heal, if ever.

In closing, Puerto Rico offers a unique setting to describe the extent of a disaster and its crippling effect on access to care. If we are diligent and gather data, we might be able to study the potential long-term effects that this tragedy could have on mental health, chronic medical problems, and cardiovascular disease.
